# Speech analysis in psychiatric disorders: experiences from the CALIBER study

**DOI:** 10.1192/j.eurpsy.2025.257

**Published:** 2025-08-26

**Authors:** G. Anmella

**Affiliations:** Bipolar and Depressive Disorders Unit, Hospital Clinic of Barcelona, Barcelona, Spain

## Abstract

**Abstract:**

Bipolar disorder (BD) is characterized by mood and cognitive fluctuations that manifest in speech patterns. Current assessments rely on subjective clinical evaluation, but advances in natural language processing (NLP) offer new opportunities for objective monitoring. This study analyzes speech from BD patients across different mood states—euthymia, mania, and depression—using structured tasks, spontaneous speech, and standardized text reading. Key acoustic, linguistic, and emotional features are extracted and correlated with clinical scales. Machine learning models are being developed to predict symptom severity and mood phase. This approach could provide reliable digital biomarkers, enhancing diagnosis, monitoring, and early relapse detection in BD. Standardized speech protocols may pave the way for international collaboration and large-scale validation.

**Disclosure of Interest:**

None Declared

